# THE IMPACT OF STROKE ON TRAJECTORIES OF LONELINESS

**DOI:** 10.1093/geroni/igad104.0260

**Published:** 2023-12-21

**Authors:** Payton Rule, Emily Willroth, Eileen Graham, Marjorie Nicholas, Robin Hattori, Tess Thompson, Lisa Connor

**Affiliations:** Washington University in St. Louis, St. Louis, Missouri, United States; Washington University in St. Louis, St. Louis, Missouri, United States; Northwestern University, Chicago, Illinois, United States; MGH Institute of Health Professions, Boston, Massachusetts, United States; Washington University School of Medicine in St. Louis, St. Louis, Missouri, United States; Washington University in St. Louis, St. Louis, Missouri, United States; Washington University School of Medicine in St. Louis, St. Louis, Missouri, United States

## Abstract

While prior research has identified loneliness as a risk factor for stroke, less is known about whether stroke may increase the risk of loneliness. We examined this question using three large representative panel studies of middle-aged to older adults in the U.S., Europe, and Israel. Specifically, we used harmonized data from the Health and Retirement Study (n=23,834, 10.3% stroke survivors), the English Longitudinal Study of Aging (n=15,779, 5.1% stroke survivors), and the Survey of Health, Aging and Retirement in Europe (n=106,480, 3.6% stroke survivors). Multilevel modeling was used to examine trajectories of loneliness across middle and older adulthood and the impact of stroke on trajectories of loneliness. Across all three samples, loneliness followed a quadratic trajectory, decreasing or remaining stable prior to age 60 and then increasing throughout older adulthood. In individuals who experienced stroke, loneliness increased more sharply after stroke onset. In follow-up analyses conducted within the subsample of stroke survivors, loneliness was higher post-stroke compared to pre-stroke. Moreover, the effect size suggests that the impact of stroke on loneliness trajectories is greater than the impact of normative developmental processes. In this talk we will discuss these results as well as the implications of this work. Specifically, these results highlight the need for loneliness prevention efforts in stroke survivors. Interventions targeting stroke survivors’ social participation and autonomy may help prevent or decrease loneliness post-stroke. Additionally, programs aimed at educating stroke survivors’ social network on ways to provide effective support may also help promote social connection in this population.

